# Tumor grade of clear cell renal cell carcinoma assessed by contrast-enhanced computed tomography

**DOI:** 10.1186/2193-1801-3-694

**Published:** 2014-11-26

**Authors:** Kousei Ishigami, Leandro V Leite, Marius G Pakalniskis, Daniel K Lee, Danniele G Holanda, David M Kuehn

**Affiliations:** Department of Radiology, University of Iowa Hospitals and Clinics, 200 Hawkins Drive, Iowa City, IA 52242 USA; Department of Urology, University of Iowa Hospitals and Clinics, 200 Hawkins Drive, Iowa City, IA 52242 USA; Department of Pathology, University of Iowa Hospitals and Clinics, 200 Hawkins Drive, Iowa City, IA 52242 USA

**Keywords:** Clear cell renal cell carcinoma, Computed tomography, Fuhrman grade, Cystic renal cell carcinoma, Growth pattern

## Abstract

The purpose of this study was to clarify the association between CT findings and Fuhrman grade of clear cell renal cell carcinoma (ccRCC). The study group consisted of 214 surgically proven ccRCC in 214 patients. Contrast-enhanced CT studies were retrospectively assessed for tumor size, cystic versus solid, calcification, heterogeneity of lesions, percentage of non-enhancing (necrotic) areas, and growth pattern. CT findings and Fuhrman grade were compared. Nineteen of 22 (86.4%) cystic ccRCC were low grade (Fuhrman grades 1-2). There was no significant correlation between tumor size and grade in cystic ccRCC (P = 0.43). In predominantly solid ccRCC, there was significant correlation between tumor size and grade (P < 0.0001). Thirty-eight of 43 (88.4%) infiltrative ccRCC were high grade (Fuhrman grades 3-4). Logistic regression showed tumor size and infiltrative growth were significantly associated with grades 3-4 (P = 0.00083 and P = 0.0059). Cystic ccRCC tends to be low grade. Infiltrative growth and larger tumor size may increase the likelihood of high grade ccRCC.

## Introduction

Clear cell renal cell carcinoma (ccRCC) is the most common subtype of RCC, accounting for approximately 70 - 80% of RCC (Leibovich et al. 2010; Teloken et al. 2009; Kim et al. 2002). It has a poorer prognosis than other subtypes of RCC, such as papillary and chromophobe RCC (Leibovich et al. 2010; Teloken et al. 2009), and its biological aggressiveness significantly affects prognosis.

The system most widely employed to classify RCC is the Fuhrman grading system, which uses the characteristics of the nuclei and nucleoli of tumor cells as its basis for grading (Fuhrman et al. 1982; Novara et al. 2007; Ficarra et al. 2005). Fuhrman grade 1 is the least aggressive type, with grade 4 being the most aggressive (Fuhrman et al. 1982). Grades 1–2 and 3–4 are classified as low and high grades, respectively (Novara et al. 2007; Ficarra et al. 2005). Higher grade tumors have an elevated risk of postoperative recurrence (Novara et al. 2007); thus, postoperative surveillance for these patients should be more rigorous. The Fuhrman grade is one of the most effective parameters used in predicting the biological aggressiveness and metastatic potential of ccRCC and papillary RCC (Novara et al. 2007; Sukov et al. 2012; Nishikimi et al. 2011), although it lacks prognostic significance for chromophobe RCC (Cheville et al. 2012).

Imaging assessment of tumor grades in RCC may aid in clinical management decisions. For example, less invasive procedures (e.g., nephron-sparing surgery and radiofrequency ablation) or close observation may be considered for low grade RCC.

There have been few previous reports citing use of magnetic resonance imaging (MRI) to compare Fuhrman grade with imaging characteristics, and the number of cases reported was relatively small (Vargas et al. 2013; Goyal et al. 2012; Rosenkrantz et al. 2010). Computed tomography (CT) is most often used for preoperative evaluation of RCC, and image quality is generally similar across institutions. Therefore, CT may be more applicable in evaluating significant numbers of cases.

The purpose of this study was to clarify the association between contrast-enhanced CT findings and Fuhrman grade of ccRCC.

## Methods

### Patient population

This retrospective study was approved by the Institutional Review Board in the University of Iowa Hospitals and Clinics and informed consent was waived.

A computerized search of the pathology and radiology database at our institution found 235 cases of clear cell renal cell carcinoma (ccRCC) that had undergone computed tomography (CT) between November 2007 and November 2012. Five cases that had only unenhanced CT were excluded, as well nine biopsy proven cases (as tumor grade from the biopsy specimen may not be identical to the actual tumor grade (Novara et al. 2007). Six cases of mixed clear cell and papillary (n = 5) and chromophobe (n = 1) RCC were also excluded because the biological aggressiveness is different. Additionally, one multiple ccRCC case was excluded because the tumor grades of each nodule were difficult to correlate. In cases of multifocal ccRCC, the largest lesion was evaluated to avoid the bias when performing the statistical analysis. The study group therefore consisted of 214 patients with 214 ccRCC that had undergone surgical resection and preoperative contrast-enhanced CT studies. One case was multilocular cystic RCC, a variant of ccRCC with an excellent prognosis (Suzigan et al. 2006; You et al. 2011; Hindman et al. 2012).

The patient group consisted of 138 males and 76 females. The ages ranged from 25 to 86 years old (mean ± standard deviation [SD]: 58 ± 13 years old). There were 102 ccRCCs found in the right kidney and 112 in the left kidney. Of these, 9 were classified as Fuhrman grade 1, 107 grade 2, 72 grade 3, and 26 grade 4 ccRCC. Fuhrman grade was determined by the attending pathologists who were blind to radiology findings. Because Fuhrman grades were obtained from pathology reports, pathologists did not re-evaluate the pathology specimens.

### Imaging analysis

Contrast-enhanced CT protocols and CT units were somewhat variable, as the study group recruited spanned a five-year period. All examinations were performed with multi-detector row CT equipped with 4, 16, or 64 detector rows. Non-ionic intravenous contrast (300, 320, 350, or 370 mgI/mL) was administered at 94 ml to 152 ml. The venous phase was obtained in 209 cases, including 94 late corticomedullary differentiation nephrographic and 115 homogeneous nephrographic phases. In the five cases where venous phase was not available, the early corticomedullary differentiation nephrographic (arterial) phase was available for review. The early corticomedullary differentiation nephrographic and delayed (excretory) phases were performed in 45 and 171 cases, respectively. Section thickness was 2, 3, or 5 mm. In addition, coronal and sagittal multiplanar reformatted (MPR) images were available for review in 190 cases.

CT studies were retrospectively reviewed by an experienced body imaging radiologist who had 18 years of experience. The reviewer knew the diagnosis of RCC but was blinded to the Fuhrman grade. MPR images were utilized if they were available. The reviewer measured the maximum diameter of the tumor size (if MPR images were available, they were utilized for the measurements). The measurements were performed twice on separate days and average data was recorded. Presence or absence of calcification was also recorded.

Tumors were classified as either cystic or predominantly solid. Cystic ccRCC was diagnosed if the tumor consisted of more than 75% of unilocular or multilocular fluid-filled non-enhancing component (Beddy et al. 2014; Koga et al. 2000; Han et al. 2004; Hartman et al. 1986) with a recognizable outer wall and/or internal septations. When an irregular solid component was circumferential, it was considered as ccRCC with central necrosis rather than cystic ccRCC. Cystic ccRCCs were classified using the Bosniak classification system (Israel & Bosniak 2005). Predominantly solid ccRCCs were classified into three types based on tumor margins: (1) well-circumscribed (expansive tumor growth with well-circumscribed and round tumor margin); (2) lobulated (lobulated tumor contour with well-defined tumor margin); or (3) infiltrative (indistinct border between the tumor and normal kidney).

Tumor enhancement for predominantly solid ccRCC was further classified as either homogenous and heterogeneous. In addition, the proportion of the non-enhancing (necrotic) area within the predominantly solid ccRCC was classified as 0 - 20%, 20 - 40%, 40 - 60%, and 60% or more.

### Statistical analysis

Fuhrman grades and imaging findings were compared using the Fisher’s exact test and the Kruskal-Wallis exact test. Tumor size was compared using the Student-t or Welch *t*-test. The correlation between the tumor grade and size was assessed by Spearman’s rank correlation. In addition, the receiver operating characteristic (ROC) curve was fit to determine the cut-off value for tumor size in the diagnosis of Fuhrman grades. Finally, logistic regression was utilized to find significant variables that would suggest Fuhrman grades 3–4 and grade 4 RCC, respectively.

All statistical analyses were performed using EZR (Saitama Medical Centre, Jichi Medical University; http://www.jichi.ac.jp/saitama-sct/SaitamaHP.files/statmedEN.html; Kanda, 2012), a graphical user interface for R (The R Foundation for Statistical Computing, Vienna, Austria, version 2.13.0). More precisely, EZR is a modified version of R Commander (version 1.6-3), designed to add statistical functions frequently used in biostatistics (Kanda 2013). All P-values were two sided, and P-values of less than 0.05 were considered statistically significant.

## Results

Of the 214 surgically resected clear cell renal cell carcinoma (ccRCC), 22 were cystic and 192 were predominantly solid. In predominantly solid ccRCC, 119 were well-circumscribed, 30 were lobulated, and 43 were infiltrative (Table 1). Of the 22 cystic ccRCC, 12 were Bosniak III, and 10 were Bosniak IV. The tumor sizes in cystic, well-circumscribed, lobulated and infiltrative ccRCC are shown in Table 2. There was no significant difference in size between cystic and well-circumscribed ccRCC. Infiltrative ccRCC was the largest followed by lobulated and well-circumscribed ccRCCs.

**Table 1:** **Fuhrman grades of clear cell renal cell carcinoma (ccRCC) in each morphology**

	Fuhrman grade				Total
	1	2	3	4	
Cystic*	3 (13.6%)	16 (72.7%)	3 (13.6%)	0 (0%)	22
Well-circumscribed	6 (5.0%)	72 (60.5%)	38 (31.9%)	3 (2.5%)	119
Lobulated†	0 (0%)	14 (46.7%)	12 (40.0%)	4 (13.3%)	30
Infiltrative‡	0 (0%)	5 (11.6%)	19 (44.2%)	19 (44.2%)	43

Note. *Cystic ccRCC has significantly lower grades than well-circumscribed ccRCC (P =0.025). †Lobulated ccRCC is higher grade than well-circumscribed ccRCC (P =0.018). ‡Infiltrative ccRCC is higher grade than lobulated ccRCC (P =0.00029).

**Table 2:** **Tumor size of ccRCC in each morphology**

	Range (cm)	Mean ± standard deviation
Cystic	1.3 - 7.7	4.1 ± 1.5
Well-circumscribed	1.1 - 13.4	3.9 ± 2.1
Lobulated†	4.2 - 14.5	8.0 ± 2.6
Infiltrative‡	3.8 – 17.2	10.7 ± 3.0

There is no significant difference in size between cystic and well-circumscribed ccRCC (P = 0.75). †Lobulated ccRCC was significantly larger than well-circumscribed ccRCC (P <0.0001). ‡Infiltrative ccRCC was significantly larger than lobulated RCC (P = 0.00023).

**Figure 1:**
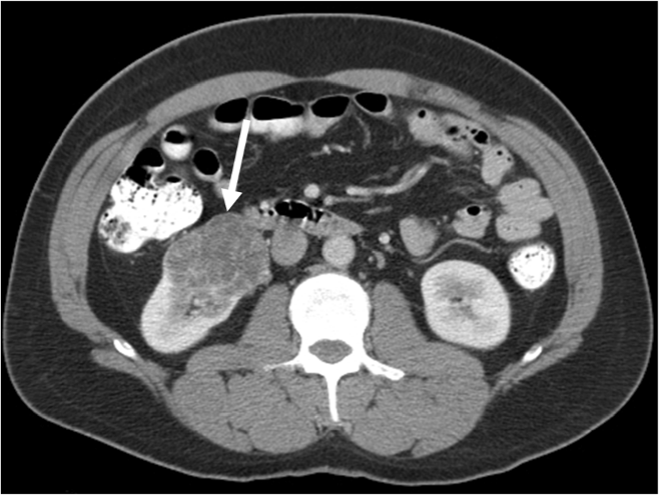
**A 43-year-old male with Fuhrman grade 2 cystic clear cell renal cell carcinoma (ccRCC).** Contrast-enhanced transverse CT image demonstrates an exophytic multilocular cystic mass in the inferior pole of the right kidney (arrow). Although multiple relatively thick enhancing septa are seen, no solid component is identified. This cystic ccRCC was categorized as Bosniak III.

**Figure 2:**
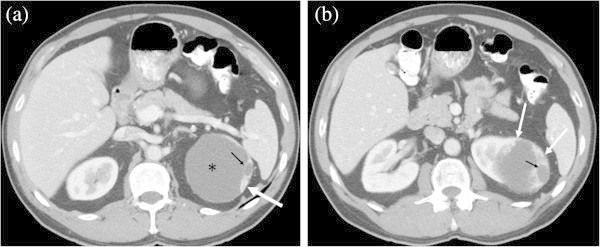
**A 58-year-old male with Fuhrman grade 3 cystic ccRCC. a**: Contrast-enhanced transverse image demonstrates a complex cystic mass in the left kidney (asterisk). An enhancing solid component with cystic change (small arrow) is noted on the left border of the cyst wall (large arrow). **b**: The section obtained through the inferior portion of the lesion below Figure 2a shows relatively larger solid enhancing components (large arrows) with cystic change (small arrow). This cystic ccRCC was categorized as Bosniak IV.

**Figure 3:**
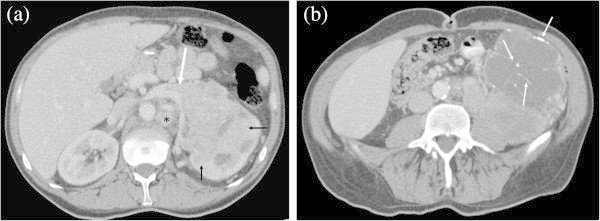
**A 43-year-old male with Fuhrman grade 4 ccRCC showing infiltrative growth. a**: Transverse contrast-enhanced CT shows a large heterogenous mass in the left kidney. The border between the tumor and normal kidney is ill-defined, representing infiltrative tumor growth (small arrows). A large arrow and asterisk show tumor thrombus in the left renal vein and left paraaortic lymphadenopathy, respectively. **b**: Transverse contrast-enhanced CT below the level of Figure 3A demonstrates the inferior portion of the tumor to be necrotic with punctate and curve linear calcifications (arrows).

**Figure 4:**
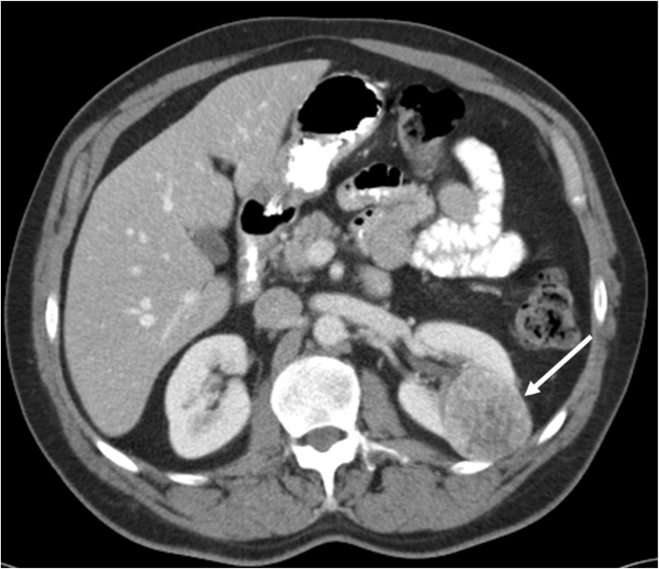
**A 45-year-old male with Fuhrman grade 3 ccRCC showing well-circumscribed tumor margin.** Contrast-enhanced transverse CT image demonstrate a predominantly solid and heterogeneously enhancing mass (arrow) in the mid portion of the left kidney. The tumor shows a well-circumscribed clear margin to the normal renal parenchyma.

**Figure 5:**
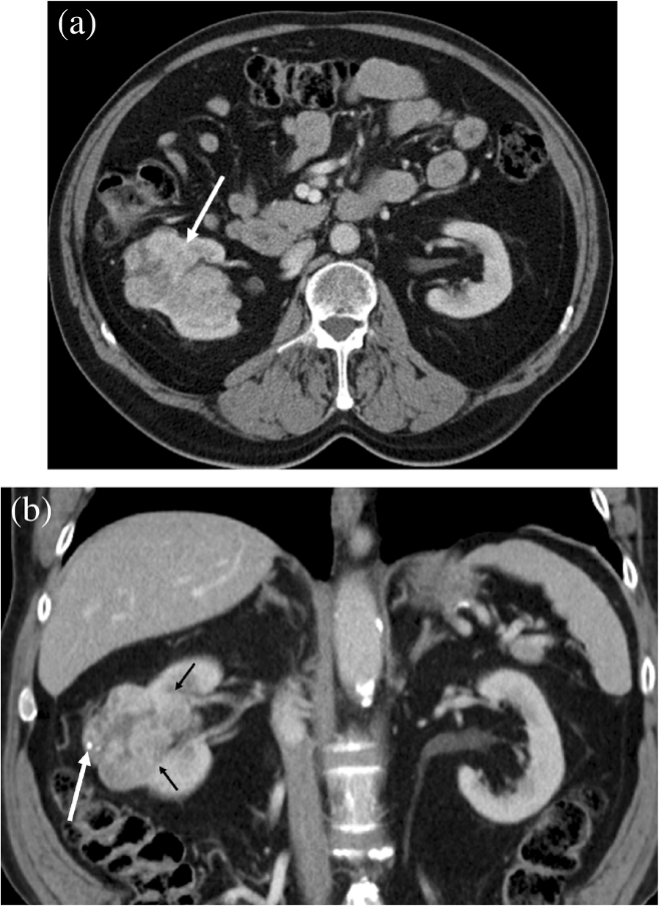
**A 74-year-old male with Fuhrman grade 2 ccRCC showing lobulated tumor margin. a**: Contrast-enhanced transverse CT image shows a heterogeneously enhancing lobulated mass in the right kidney (arrow). **b**: Coronal reformatted image shows lobulated tumor contour and well-defined tumor margin to the normal renal parenchyma (small arrows). Note calcification in the tumor (large arrow).

Tumor morphology and Fuhrman grade are shown in Table 1. Nineteen of 22 (86.4%) cystic ccRCC were low grade (Fuhrman grades 1 or 2) (Figure 1) (Tables 1 and 2). All of the three cystic ccRCC with grade 3 were Bosniak IV (Figure 2). There was a significant difference in tumor grade between cystic ccRCC and well-circumscribed ccRCC (P = 0.025). Thirty-eight of 43 (88.4%) infiltrative ccRCC were high grade (Fuhrman grades 3 or 4) (Figure 3). There were significant differences in tumor grades between well-circumscribed (Figure 4) and lobulated ccRCC (Figure 5) (P = 0.018), and between lobulated and infiltrative RCC (P = 0.00029).

**Figure 6:**
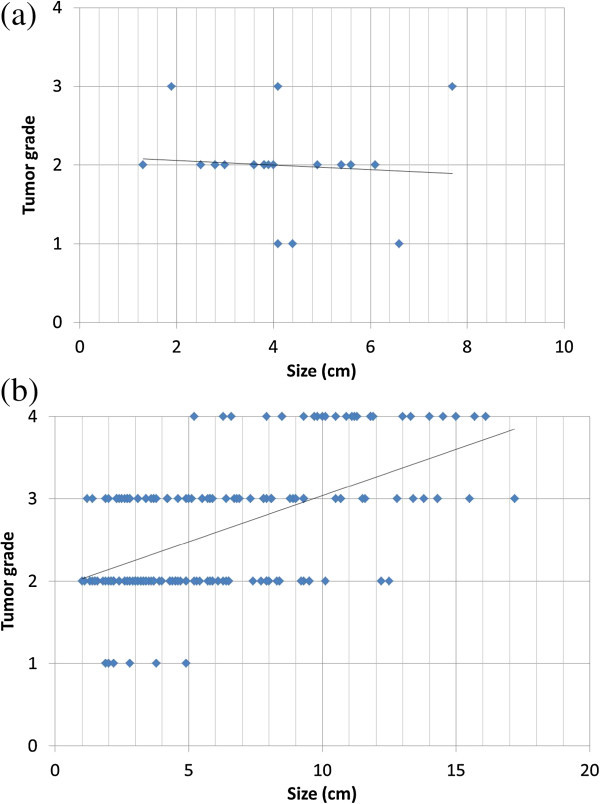
**Relationship between tumor size and Fuhrman grade in cystic ccRCC (a) and predominantly solid ccRCC (b). a**: The X-axis is tumor size (cm), and the Y-axis is Fuhrman grade (1–4). The figure shows scatter graph and the fitted line. In cystic ccRCC, there is no significant correlation between tumor size and Fuhrman grade. Spearman’s rank correlation Rho was -0.179 (P =0.43). **b**: Scatter graph and the fitted line of predominantly solid ccRCC. There is significant correlation between tumor size and Fuhrman grade. Spearman’s rank correlation Rho was 0.51 (P <0.0001).

In cystic ccRCC, there was no significant correlation between tumor size and Fuhrman grade. Spearman’s rank correlation Rho was -0.179 (P = 0.43, Figure 6a). By contrast, in predominantly solid ccRCC, there was significant correlation between tumor size and Fuhrman grade. Spearman’s rank correlation Rho was 0.51 (P <0.0001, Figure 6b).

Receiver operating characteristic (ROC) curves showed the cut-off value of 5.0 cm in the diagnosis of Fuhrman grades 3-4 ccRCC. The sensitivity, specificity, and the area under the ROC curve were 69.4%, 69.8%, and 0.740, respectively.

Other imaging findings including calcification, heterogeneity of the lesions, and the percentage of non-enhancing (necrotic) area within the predominantly solid ccRCC are summarized in Table 3. The univariate analysis of imaging findings and the Fuhrman grades are shown in Table 4. Univariate analysis showed that calcification and ≥60% of non-enhancing area were significantly more common in high grade ccRCC than in low grade ccRCC. There was no significant difference between heterogeneous and homogeneous ccRCC. In addition, the mean size of ccRCC with calcification was larger than that of ccRCC without calcification (10.0 ± 3.9 cm vs. 5.1 ± 3.0 cm, P <0.0001) (Figure 3).

**Table 3:** **Imaging findings of ccRCC**

		Fuhrman grade				Total
		1	2	3	4	
Homogeneous		1	7	8	0	16/192 (8.3%)
Heterogeneous		5	84	61	26	176 (91.6%)
Calcification		0	10	13	11	34/214 (15.9%)
Non-enhancing area	< 20%	3	30	20	7	60/192 (30.9%)
	≥ 20%, <40%	1	25	18	9	53/192 (27.3%)
	≥ 40%, <60%	1	22	12	2	37/192 (19.1%)
	≥ 60%	1	14	19	8	42/192 (21.6%)

Note. The data show the number of the lesions. The data in the parentheses show the percentage. The heterogeneity of the lesions (homogeneous or heterogeneous) and the percentage of non-enhancing (necrotic) area were evaluated in the predominantly solid ccRCC (n = 192). The presence or absence of calcification was evaluated in total ccRCC cases (n = 214).

**Table 4:** **Univariate analysis of imaging findings of ccRCC and Fuhrman grades**

	Grades 1-2	Grades 3-4	P-value
Calcification* +/- (n = 214)	10/106	24/74	0.0023
Tumor heterogeneity Homo/Hetero (n = 192)	8/89	8/87	1.0
Non-enhancing area^†^ ≥60%/<60% (n = 192)	15/82	27/68	0.036

Note. The data show the number of the lesions. The heterogeneity of the lesions (homogeneous or heterogeneous) and the percentage of non-enhancing (necrotic) area were evaluated in the predominantly solid ccRCC (n = 192). The presence or absence of calcification was evaluated in total ccRCC cases (n = 214).

*Calcification is significantly more common in grades 3–4 than grades 1–2. ^†^Non-enhancing area 60% or more within the predominantly solid clear cell renal cell carcinoma is significantly more common in grades 3–4 than grades 1–2.

Logistic regression analysis showed the tumor size and infiltrative growth were significant variables to correlate with Fuhrman grades 3–4 ccRCC (P = 0.00083 and P = 0.0059, respectively, Table 5). Calcification, lobulated contour, and ≥60% non-enhancing area were not significant variables for Fuhrman grades 3–4.

**Table 5:** **Multivariate (logistic regression) analysis in the diagnosis of Fuhrman grades 3–4 ccRCC**

	Odds ratio	95% CI	P-value
Size*	1.2	1.1 – 1.4	0.00083
Infiltrative^†^	4.9	1.6 – 15.4	0.0059
Calcification	0.9	0.3 – 2.5	0.78
Lobulated	4.9	0.9 – 27.7	0.069
Non-enhancing area ≥60%	1.7	0.7 – 3.9	0.20

Note. CI = confidence interval. The data were analyzed in total 214 ccRCC cases.

*^†^Tumor size and infiltrative growth are significantly associated with grades 3–4 RCC.

## Discussion

Clear cell renal cell carcinoma (ccRCC) is the most common subtype of RCC, followed by papillary and chromophobe RCCs. Characteristics of ccRCC are possible to differentiate from papillary and chromophobe RCCs on diagnostic imaging (Kim et al. 2002), although it is not 100% accurate. Because papillary RCC is generally more indolent than ccRCC (Leibovich et al. 2010; Teloken et al. 2009), and Fuhrman grade lacks prognostic significance for chromophobe RCC (Cheville et al. 2012), papillary and chromophobe RCCs were not considered in this study. Oncocytoma, a less common benign renal tumor, was beyond the scope of this study because it can be difficult to prospectively differentiate it from ccRCC on diagnostic imaging. For these reasons, we focused solely on the imaging findings of ccRCC and their relationship to Fuhrman grade.

In our study, cystic ccRCC was more likely (88.5%) to be low grade (Fuhrman grades 1–2), and no correlation was found between the size of cystic ccRCC and Fuhrman grade (Figure 6a). These results were in keeping with previous studies that showed cystic RCC to have a better prognosis than solid RCC (Koga et al. 2000; Han et al. 2004) and can be explained by the fact that fewer malignant cells are present in cystic RCC than predominantly solid RCC. Our results suggest that less invasive procedures such as nephron-sparing surgery or close observation may be considered for cystic RCC.

Our study did include a small percentage of high grade (Fuhrman grades 3–4) cystic ccRCC, although it was not statistically significant by multivariate analysis. RCC with cystic necrosis has the worst prognosis among the cystic RCCs (Han et al. 2004; Hartman et al. 1986), and one previous study showed the presence of tumor necrosis correlated with aggressive histology (Beddy et al. 2014). Distinguishing cystic ccRCC from ccRCC with extensive central necrosis can be a problematic in some cases, although we did not evaluate interobserver variability on this study. When ccRCC is equivocal for solid or cystic type, it is recommended that the type be considered predominantly solid to avoid underestimation.

The increased likelihood of high grade tumors in infiltrative ccRCC (88.4%) may reflect biological tumor aggressiveness (Figure 3). For example, RCCs with aggressive histology, such as type 2 papillary RCC (Yamada et al. 2008; Rosenkrantz et al. 2013) and collecting duct RCC (Yoon et al. 2006), commonly show an infiltrative appearance on imaging. Similarly, it has been reported that a histopathological finding of infiltrative growth was a factor indicating poor prognosis in ccRCC (Nishikimi et al. 2011). Our results suggest that careful post-operative surveillance may be necessary for RCC with infiltrative growth on imaging findings.

The association of larger tumor size with higher grade, stage and metastasis has been described in previous reports (Umbreit et al. 2012; Zhang et al. 2012). However, it should be recognized that there can be some overlap in CT findings between Fuhrman grades 2 and 3 ccRCC.

Calcification and 60% or more non-enhancing area were commonly present in large tumors, which may explain why these findings were not significant by multivariate analysis in our study. In addition, one previous study showed that decreased tumor vascularity was associated with high grade RCCs (Vargas et al. 2013). Because the CT protocols of our study group varied somewhat, our ability to evaluate tumor enhancement may have been limited. Further study to clarify the association between tumor neo-vascularity in ccRCC and tumor grade may be necessary.

The limitations of this study include 1) a retrospective study at a single institution, 2) non-uniform CT acquisition techniques, 3) no correlation of CT findings with histopathology results except Fuhrman grade of ccRCC, 4) a retrospective review of CT findings by a single experienced radiologist without assessment of interobserver variability, 5) inclusion of surgically proved clear cell RCC only and exclusion of other primary renal neoplasms, and 6) Fuhrman grades obtained from pathology reports without re-review.

In summary**,** cystic ccRCC tended to be low grade (Fuhrman grades 1–2), and tumor size did not correlate with tumor grade. For predominantly solid ccRCC, infiltrative growth and larger tumor size may increase the likelihood of high grade ccRCC (Fuhrman grades 3–4).
